# Hemophagocytic Lymphohistiocytosis in Association With Neuroblastoma Amplified Sequence (NBAS) Gene Variants: A Report of a Rare Case

**DOI:** 10.7759/cureus.69690

**Published:** 2024-09-19

**Authors:** Siddhi Gawhale, Sampada Tambolkar, Parag Tamhankar, Balasubramanya S Tandur, Sarita Verma

**Affiliations:** 1 Pediatrics, Dr. D.Y. Patil Medical College, Hospital and Research Center, Dr. D.Y. Patil Vidyapeeth (Deemed to be University), Pune, IND; 2 Paediatrics, Dr. D.Y. Patil Medical College, Hospital and Research Center, Dr. D.Y. Patil Vidyapeeth (Deemed to be University), Pune, IND; 3 Pediatric Oncology, King Edward Memorial Hospital, Pune, IND

**Keywords:** hemophagocytic lymphohistiocytosis (hlh), immunomodulation, natural killer, nbas gene mutation, primary hlh, stem cell applications

## Abstract

Hemophagocytic lymphohistiocytosis (HLH) is a multisystem involvement, hyperinflammatory state with rapid progression and a poor outcome. However, HLH may rarely present with signs and symptoms isolated to the central nervous system (CNS). Thus, we discuss this case, which presented with CNS symptoms and worsened over time with multisystem involvement, an inflammatory storm, and required immunomodulation. Whole exome sequencing performed on genomic DNA extracted from peripheral blood showed a novel finding that the patient was likely compound heterozygous for the following two novel variants of uncertain significance in the neuroblastoma amplified sequence (NBAS) gene (chr2:g.15461289C>T) or c.2251G>A (p.Asp751Asn) on Exon 21 and (chr2:g.15467334A>G) or c.2092T>C (p.Tyr698His) on Exon 19 (genomic coordinates in the GRCh37 format, transcript ID: NM_015909.4). The NBAS gene is needed for cytotoxic degranulation in natural killer (NK) cells and mutation of which dysregulates lytic vesicle transport, thus leading to the hyperinflammatory state. To the best of our knowledge and according to the available literature, this NBAS gene is a rarely documented cause of primary HLH.

## Introduction

Hemophagocytic lymphohistiocytosis (HLH) causes abnormal immune system activation and tissue damage. Large amounts of cytokine secretion led to tissue breakdown and organ dysfunction [1]. The absence of downregulation of lymphocytes and activated macrophages due to the failure of natural killer (NK) cells and cytotoxic T-cells to eliminate them is one of the proposed reasons for the hyperinflammatory state [2,3]. HLH may be classified as primary and secondary or familial and acquired. Primary HLH is due to gene mutations, and secondary HLH is present without a known genetic mutation but as a response to an infective trigger or any malignancy. However, any illness may trigger and flare up an underlying genetic mutation and have overlapping features of both primary and secondary HLH, creating a diagnostic dilemma. Therefore, a workup should be done for primary HLH because this is a more robust treatment that, if not done, has a guarded prognosis. HLH has clinical features similar to those of sepsis, septic shock, and other systemic inflammatory states.

The Familial Hemophagocytic Lymphohistiocytosis Study Group established the diagnostic criteria in 1991 [4] for children under 15 years old. The first treatment protocol for HLH was introduced in 1994 [5] and was later updated in 2004. To diagnose HLH, five out of the following eight criteria must be met [6]: (1) fever (≥38.5 °C); (2) splenomegaly; (3) bicytopenia, including hemoglobin levels below 9 g/dL, thrombocytopenia under 100,000/µL, and an absolute neutrophil count below 1,000/µL; (4) hypertriglyceridemia (>265 mg/dL), reduced fibrinogen (≤150 mg/dL); (5) presence of hemophagocytes in the bone marrow aspiration, liver, spleen, or lymph nodes; (6) reduced NK cell activity (<10 lytic units); (7) raised ferritin levels (≥500 µg/L); and (8) soluble CD25/IL-2 excess (≥2,400 U/mL). These criteria may occur in other pathological states, and thus Daver et al. [7] added other variables to improve the diagnostic accuracy for HLH, especially developed for cases with potential malignancy. These criteria include (1) hepatomegaly; (2) hypoalbuminemia (<3.5 g/dL); (3) deranged coagulation profile (prothrombin time (PT) ≥ 1.5 times upper normal limit, partial thromboplastin time ≥ 1.5 times upper normal limit and/or Ddimer ≥ 10.0 µg/mL); (4) raised hepatic enzymes, ≥2.5 times the upper limit of normal; (5) renal failure (50% increase in creatinine from the baseline); (6) absolute monocyte more than 1,000/µL; (7) raised β2 microglobulin (≥2 mg/L); and (8) raised lactate dehydrogenase (≥2.5 times upper normal limit). Primary HLH can be categorized into two types: familial HLH, an autosomal recessive disorder caused by various mutations in the NK/T cell cytotoxic pathway, and HLH associated with primary immunodeficiency syndromes [8]. These immunodeficiency syndromes include Hermansky-Pudlak syndrome type 2, Chédiak-Higashi syndrome, X-linked lymphoproliferative syndrome (XLP), and Griscelli syndrome type 2 [9].

Whole exome sequencing in the present study revealed neuroblastoma amplified sequence (NBAS) gene involvement, thus enabling the diagnosis of primary HLH. The NBAS gene encodes a protein found in neuroblastoma cells and functions as a component of an endoplasmic reticulum (ER) tethering complex interacting with several proteins. NBAS messenger RNA is highly expressed in connective tissues, eye, brain, and spinal cord. Recently, NBAS has been identified as a member of a subunit of the syntaxin 18 complex, a membrane transport; depletion of NBAS causes the redistribution of Golgi recycling proteins and defect in protein glycosylation, thus proving the role of NBAS in Golgi-to-ER transport and is inherited in an autosomal recessive pattern. It has been studied that the loss of function mutation of NBAS is associated with the pathogenesis of short stature, optic nerve atrophy, and Pelger-Huet anomaly of granulocytes (SOPH syndrome) in the Siberian Yakut population, and recurrent acute hepatic failure in infancy in the European population [10].

As it is crucial to distinguish between primary and secondary HLH, we describe here a case that presented with neurological signs with rapid deterioration, which was linked to primary HLH that is associated with the NBAS gene variant.

## Case presentation

A four-month-old boy presented to our emergency department after being referred from another hospital and was mechanically ventilated. He was admitted there with a high-grade fever two days prior to presentation to that hospital, associated with an episode of uprolling of the eyeballs, frothing from the mouth, and tonic-clonic movements of all four limbs. He was treated for the same. The cerebrospinal fluid examination was within normal limits, and the viral polymerase chain reaction panel was negative. On day four of admission, he had deteriorating sensorium, poor activity, and disordered breathing. He also had signs of poor perfusion, for which he was started on vasoactive support and electively intubated. He had raised C-reactive protein levels with pancytopenia and persistent fever and was evaluated for the possibility of HLH. He had decreased fibrinogen levels, elevated triglycerides, hyperferritinemia (>2,000 units), and elevated LDH (>800 units). He also had hypoalbuminemia, as shown in Table 1. Dengue ELISA was negative.

**Table 1:** Laboratory investigations TLC: total leucocyte count; INR: international normalized ratio RBC: red blood cell; CRP: C-reactive protein, LDH: lactate dehydrogenase; CSF: cerebrospinal fluid

Parameter (reference range)	Lab value
Hemoglobin (12.8-16 g/dL)	8
TLC (4000-9100/µL)	6600
Neutrophils (40-70%)	44%
Lymphocytes (20-40%)	51%
Platelet count (150,000-410,000/µL)	78000
Packed cell volume (37.3-47.7%)	24
INR (1-1.2)	1.68
CRP (<2 mg/L)	44
Erythrocyte sedimentation rate (up to 10 mm/hr)	48
Alanine aminotransferase (7-55 U/Lt)	55
Aspartate aminotransferase (8-60U/Lt)	24
Creatinine (0.35-0.86 mg/dL)	0.32
Serum Sodium (135 -145 mEq/L)	140
Serum Potassium (3.5 -5.5 mEq/L)	3.8
Serum Albumin (3.5 -5.5 g/dL)	2.9
CSF Protein (15 -60 mg/dL)	39
Glucose (50-80 mg/dL)	75
Total cell count (0-5)	4 (100% lymphocytes)
CSF Pyruvate (0.7 – 1.7mM )	0.75
CSF Lactate (1.10 - 2.4mM)	2.2
Serum Fibrinogen levels (200-400mg/dL)	113
Serum Triglycerides (100-150 mg/dL)	432
Serum LDH ( 140-280 U/L)	876
Serum Hyperferritinemia (30- 400 ng/mL)	2880

**Figure 1:**
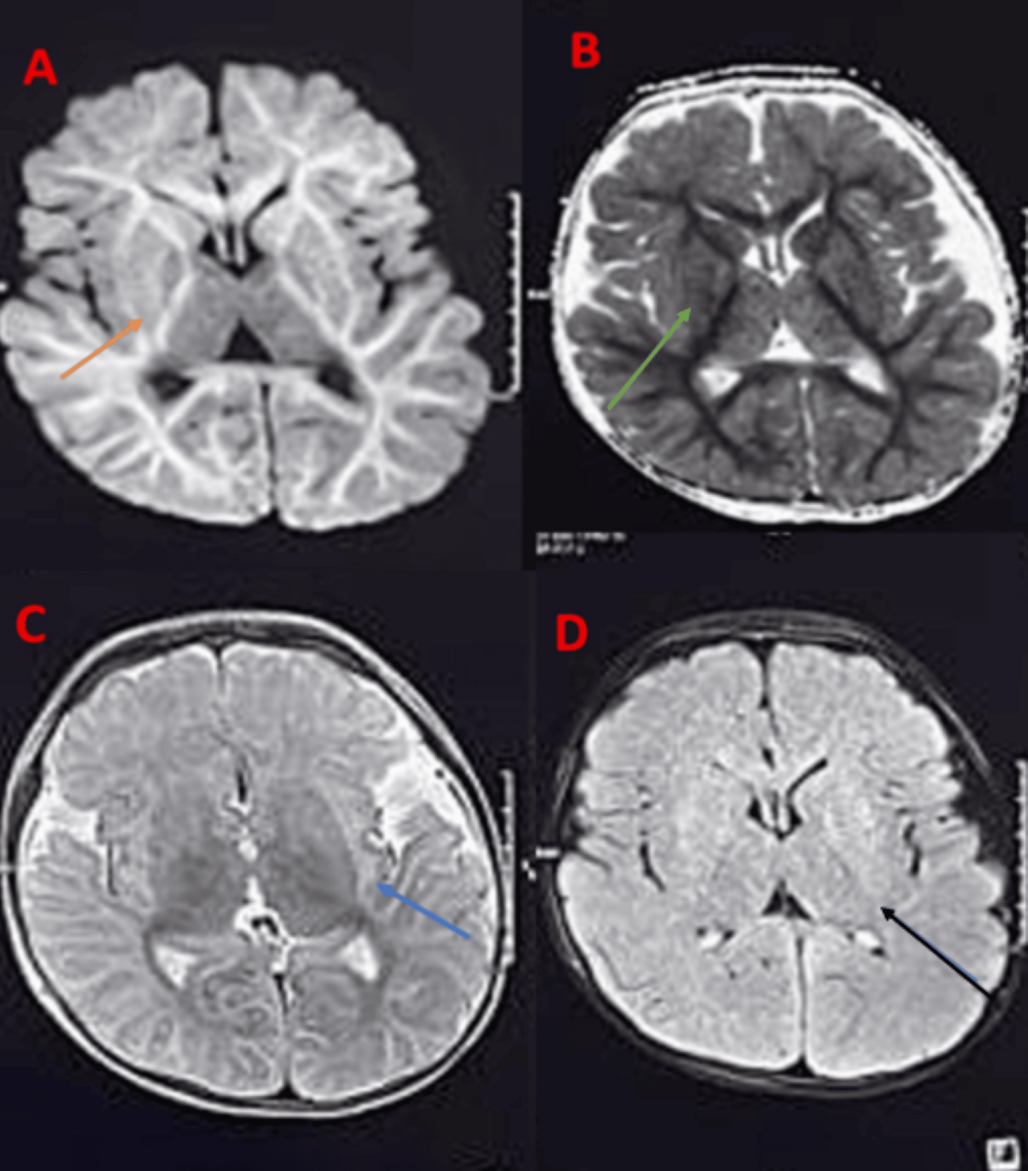
MRI brain A: diffusion-weighted imaging (orange arrow). B: apparent diffusion coefficient (green arrow), showing diffusion restriction involving the entire subcortical white matter, corpus callosum, centrum semiovale, internal and external capsule. C: T2 axial section, showing external capsule hyperintensity (blue arrow). D: FLAIR axial section, showing hyperintensities in the corpus callosum and internal and external capsule (black arrow)

He was then administered intravenous pulse-dose methylprednisolone at 30 mg/kg/day for five days and IVIG at 2 gm/kg as immunomodulation, considering HLH. An oral steroid (prednisolone at 2 mg/kg/day) was administered after the pulse steroid and was tapered over two weeks. Packed cell volume was transfused under diuretic cover. The child improved clinically, and HLH markers had a decreasing trend. Functional cardiac 2D echo was essentially normal, with a normal cardiac output. Suspecting Inborn errors of metabolism, tandem mass spectrometry and gas chromatography-mass spectrometry (TMS-GCMS) were within normal limits. The child had multiple episodes of seizures on day nine of admission while on ventilatory support and was treated with multiple antiepileptics and a midazolam infusion. MRI brain showed extensive bilateral symmetrical FLAIR hyperintensities and DWI restriction seen in corona radiata, subcortical white matter, centrum semiovale, corpus callosum, and internal capsule, as shown in Figure 1.

**Figure 2:**
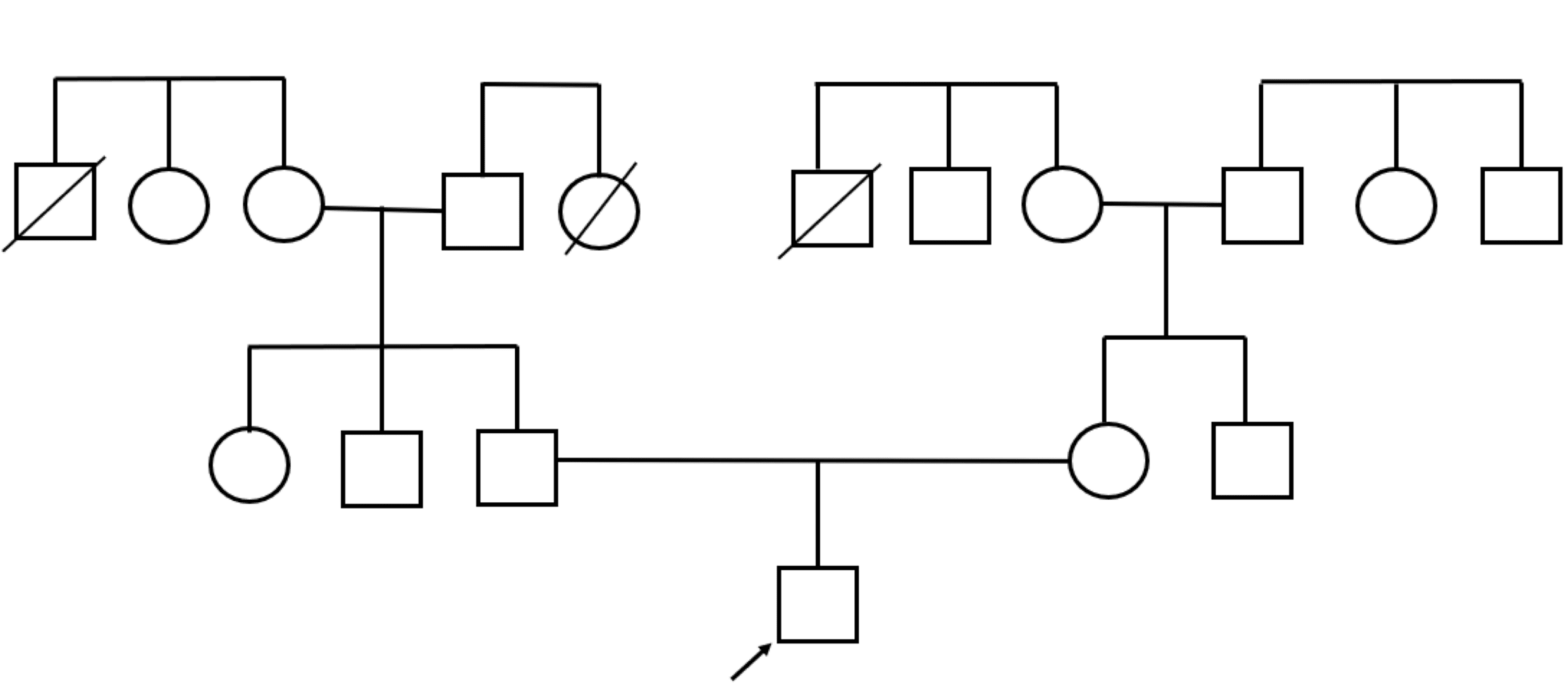
Family pedigree The squares show male members, while the circle show female members. The oblique line on the square or circle represent deceased members. The arrow represents our case.

He had no significant bi­­­­rth history, the child was born of non-consanguineous marriage, and both parents had no significant family history (see Figure 2, family pedigree). He had no prior admissions and had developmental milestones appropriate for his age.

The child responded well to immunomodulation, and vasoactive support was tapered off. Antiepileptics were also tapered with EEG monitoring, and ventilatory support was weaned. He was successfully extubated, and feeds were established. Primary Immunodeficiency disorder workup including lymphocyte subset analysis showed a normal lymphocyte subpopulation except for borderline low absolute T lymphocytes (CD3+/CD19-) 1688 cells/ μL (1900-5900 cells/ μL) and low absolute Th lymphocyte (CD3+/CD4+) 608 cells/μL(1400-4300 cells/ μL) and borderline low memory B-cells 2.81% (3.5-12.2%), nitroblue tetrazolium test burst cell 98% (95-100%) and perforin expression on CD56+ NK cells was normal. Hence, the above reports were not consistent with primary immunodeficiency. Further genetic studies suspecting the possibility of primary HLH were sent. Whole exome sequencing performed on genomic DNA extracted from peripheral blood showed that the patient was likely compound heterozygous for the following two novel variants of uncertain significance in the NBAS gene (chr2:g.15461289C>T) or c.2251G>A (p.Asp751Asn) on Exon 21 and (chr2:g.15467334A>G) or c.2092T>C (p.Tyr698His) on Exon 19 (genomic coordinates in the GRCh37 format, transcript ID: NM_015909.4). Parental testing by Sanger sequencing to confirm these variants in a compound heterozygous state was advised. The variant p.Asp751Asn in the NBAS gene is a missense variant aspartic acid to asparagine and satisfies the following American College of Medical Genetics and Genomics (ACMG) pathogenicity guidelines: PM2 (pathogenic moderate-extremely low-frequency in the gnomAD population database, which was 0.000007 and no homozygotes) and BP4 (benign supporting-computational tools support a benign effect on the gene). Franklin classification of this variant is a variant of uncertain significance. The variant p.Tyr698His in the NBAS gene is a missense variant tyrosine to histidine and satisfies the following ACMG pathogenicity guidelines: PM2 (pathogenic moderate-extremely low frequency in the gnomAD population database, which was 0.0002 and no homozygotes) and BP6 (benign supporting-Clinvar database reports suggest that the findings are likely benign; however, the necessary evidence for conducting an independent evaluation is not available). Franklin classification of this variant is a variant of uncertain significance.

## Discussion

Our case was unusual since it started predominantly with CNS involvement and then evolved to multiple organ involvement with rapid progression. HLH was the focus of the clinical and laboratory workup because of the sudden, inexplicable decline. Through immunomodulation, the patient's clinical course was improved, allowing a better outcome. Immunodeficiency was ruled out, and whole exome sequencing helped in the diagnosis. CNS involvement is observed in up to 75% of HLH cases [11], which present with symptoms such as encephalopathy, ataxia, hemiplegia, cranial nerve dysfunction, altered mental status, seizures, meningitis, or general irritability. Although uncommon, there are cases where CNS symptoms occur without systemic involvement, referred to as cerebral HLH [12,13]. Imaging may show characteristics such as enhancing nodular parenchymal lesions, leptomeningeal enhancement, demyelination, and atrophy [14]. HLH should be considered in instances of abrupt onset and progressive unexplained systemic inflammatory response syndrome (SIRS), which may present with symptoms such as fever, hepatosplenomegaly, coagulopathy, jaundice, generalized lymphadenopathy, malaise, and cytopenias. Cases of neonatal HLH manifesting solely as acute hepatic failure have been documented. The primary aim of HLH treatment is to control the overactive immune response and prevent additional tissue damage.

The revised treatment guidelines from the Second International Conference of the Histiocyte Society in 2004 suggest an eight-week induction therapy that includes corticosteroids, etoposide, and cyclosporine A as the core approach for managing HLH [15]. Corticosteroids help to manage the cytokine storm, etoposide prevents cell division and proliferation, and cyclosporine A inhibits T-cell activation. In certain cases, stem cell transplantation is recommended and can significantly enhance three-year survival rates from nearly 0% to 50% in familial HLH cases [16], with better results seen with reduced-intensity regimens [17]. Whole exome sequencing in our case suggested primary HLH related to NBAS gene variants. We performed a literature review of NBAS gene-associated cases with immunodeficiency or HLH. Ricci et al. [18] identified a child with homozygous NBAS gene variant p.Pro650Ser in a newborn screening program for immunodeficiency. Clinical features in the child included low birth weight (1,520 gm), intolerance for enteral feeding, elevated liver transaminase, eczema, rotavirus enteritis, *Klebsiella pneumoniae *urinary tract infection, methicillin-resistant *Staphylococcus aureus *sepsis, lymphocytopenia, eosinophilia, low IgG, absent IgA, IgM, higher levels of IgE, normal expression of T-cell excision circles, complete absence of K-deleting recombinant circles, absent CD19+ cells, and low CD8+ cells and NK cells. Lenz et al. [19] showed that patients with NBAS gene variants have multisystem disease including an immunodeficiency with quantitative and qualitative NK cell and B cell deficiency. Their patients had the following NBAS gene variants: p.Ile187del, p.Ser230Glnfs*4, p.Ser414Phe, p.Ile984Leufs*8, p.Glu2080Ter, p.Cys426Trp, p.Val1528Glyfs*2, p.Pro650Ser, p.Leu903Arg, p.Glu943Ter, p.His940Pro, p.Ser1178Arg, c.1342-6A>G, p.Arg1914His, p.Ala1921Pro, p.Trp1850Glyfs*32, p.Gln2322Hisfs*18, p.Leu202del, and p.Leu1055Pro. These patients had recurrent infections such as recurrent pneumonia, urinary tract infections, tonsillitis, gastroenteritis, otitis media, meningitis, and endocarditis. Patients had reduced levels of IgA, IgM, and IgG, and those who received regular immunoglobulin injections had a lowered rate of infections. Patients had impaired response to vaccination showing defects in adaptive humoral immunity. Patients showed low CD19+ cells and CD56+ NK cells. They also showed that the NK cells were less responsive to target cell stimulation in terms of cytolytic degranulation. They demonstrated that cytokine interleukin -2 (IL-2) induced activation can improve the degranulation in NBAS gene-deficient NK cells in response to target stimulation, thereby proposing IL-2 as a therapeutic option. Xiaoman Bi et al. [20] identified NBAS gene variants in 2.11 % of their HLH patient cohort after exome/genome trio analysis. The NBAS gene variants identified in this study included p.P454R, p.R1073C, p.T1097M, p.P1474L, p.Y1495H, p.K1517E, p.E1521Q, p.M1743V, p.Q2232E, and p.E2269K.

The NBAS gene is involved in the Golgi body to endoplasmic reticulum reverse transport system and is essential to the NK cell cytolytic degranulation pathway. Xiaoman Bi et al. showed that the knockdown of NBAS in an NK cell model resulted in a lower number of cytotoxic vesicles near the Golgi body and impairment of the lytic granule polarization. Juhua Ji et al. [21] identified compound heterozygous variants in NBAS genes c.939_939delGC (p.Arg313Profs*13) and p.Cys448Arg in a patient with recurrent fever-triggered liver failure, normal serum immunoglobulin profile, and increased cytotoxic CD8+ cells but normal CD19+ B cells. Uncontrolled activation of CD8+ cytotoxic lymphocytes is one of the proposed mechanisms of HLH, thus supporting the role of NBAS gene variants in the pathogenesis of HLH. The prognosis for genetic HLH is poor without treatment, with a median survival time between one and two months and a survival rate of less than 10% over three years [22]. The primary differential diagnosis for HLH is SIRS. In addition, HLH can be misdiagnosed as neonatal hemochromatosis [23] or a metabolic crisis in infants who present with severe organomegaly or elevated triglyceride levels. Infections can also clinically resemble HLH, although hemophagocytosis should not be evident in the bone marrow. Findings from lymph node biopsies may be similar to those seen in malignant lymphomas [24]. Hence, timely diagnosis, ruling out differentials, and aggressive treatment can help improve outcomes by halting the hyperinflammatory process.

## Conclusions

Overlapping traits led to an underdiagnosis of HLH in the past. Nonetheless, as a result of numerous case reports, it is now acknowledged as one of the crucial diagnoses for cases that are rapidly deteriorating and involving multiple systems. Examining each suspected case of HLH for cancer, infectious diseases, or genetic abnormalities is important. The availability of genetic diagnostics and recent developments in our understanding of the pathophysiology of genetic HLH have improved the available treatment regimen and increased the survival rate. The prognosis is poor in these situations, so screening parents and index cases for immunodeficiency is crucial to improving outcomes for the following generation. To ascertain whether acquired HLH is a unique illness or just the conclusion of an immune system, more investigation is required. Further research on the association of the variants in the NBAS gene (viz. p.Asp751Asn and p.Tyr698His) with HLH is needed to determine their clinical significance.
